# Letter to the Editor: Same-day discharge vs. inpatient stay in laparoscopic sleeve gastrectomy: a systematic review and meta-analysis

**DOI:** 10.1097/JS9.0000000000003287

**Published:** 2025-08-22

**Authors:** Chuanwei Zhao, Wenzhou Yang

**Affiliations:** Department of Cardiology, The Second People’s Hospital of Baoshan City, Baoshan, Yunnan, China

Mobarak *et al* synthesize evidence from >18 000 patients and show that same-day discharge (SDD) after laparoscopic sleeve gastrectomy yields comparable major-complication and re-operation rates to conventional overnight stay, although a modest uptick in 30-day readmission is observed (pooled OR 1.18, 95% CI 1.01–1.39)^[1]^. Their work is timely: SDD programs are expanding under enhanced recovery after surgery (ERAS) pathways and, if safe, could alleviate bed pressures and reduce costs without compromising outcomes. This need for transparent methodology and rigorous external validation is consistent with the 2025 TITAN Guidelines on reporting advanced analytics and artificial intelligence studies in surgery, which emphasize complete disclosure of model development and validation before clinical adoption^[2]^.

Three issues merit closer scrutiny. First, clinical heterogeneity across the 11 included cohorts is substantial: some centers restricted SDD to ASA I–II patients with body mass index <50 kg/m^2^, whereas others accepted higher-risk profiles^[3]^. Large registry analyses confirm that comorbidity burden (e.g., renal insufficiency, prior myocardial infarction, and smoking) modifies SDD risk, with Black race and Roux-en-Y bypass (RYGB) demonstrating higher complication odds^[4]^. Pooling such dissimilar populations may attenuate true between-group differences.

Second, process-of-care variables are incompletely reported. Dedicated ERAS protocols, multimodal analgesia, and mandatory postoperative call-backs halve unplanned emergency-department visits after SDD sleeve gastrectomy^[5]^, yet only 4 of the 11 studies detailed their perioperative pathways^[1]^. Future meta-analyses should stratify outcomes by ERAS compliance to separate surgical factors from program design.

Third, prospective data are urgently needed. Remote wireless vital-sign monitoring has enabled safe SDD in selected bariatric patients with <1% complications and 0% mortality rates at 30 days^[6]^, suggesting that technology-assisted surveillance can substitute for inpatient observation. A pragmatic, multicenter randomized trial comparing standard SDD with “telemonitoring-assisted SDD” and inpatient stay – using uniform inclusion criteria and core outcome sets – would clarify the incremental value of each strategy.

In summary, the meta-analysis by Mobarak *et al* advances the discussion on ambulatory bariatric surgery, but clearer patient-selection algorithms, reporting of ERAS elements, and prospective validation with digital monitoring are pivotal before widespread adoption.

## Data Availability

This is a commentary article. No new data were generated or analyzed; therefore, data availability is not applicable.
